# TOR1B: a predictor of bone metastasis in breast cancer patients

**DOI:** 10.1038/s41598-023-28140-y

**Published:** 2023-01-27

**Authors:** Minh Nam Nguyen, Salima Akter, Hajara Akhter, Shahina Ansary, Sunhee Han, Yoonhwa Shin, Joohun Ha, Insug Kang, Sung Soo Kim, Tae Gyu Choi

**Affiliations:** 1 Department of Biomedical Engineering, School of Medicine, Vietnam National University Ho Chi Minh City (VNU-HCM), Ho Chi Minh City, 700000 Vietnam; 2 Research Center for Genetics and Reproductive Health (CGRH), School of Medicine, National University HCMC, Ho Chi Minh City, 700000 Vietnam; 3 Vietnam National University Ho Chi Minh City (VNU-HCM), Ho Chi Minh City, 700000 Vietnam; 4 Department of Biochemistry and Molecular Biology, School of Medicine, Kyung Hee University, Seoul, 02447 Republic of Korea; 5 Department of Medical Biotechnology, Bangladesh University of Health Sciences, Dhaka, 1216 Bangladesh; 6 Biomedical and Toxicological Research Institute, Bangladesh Council of Scientific and Industrial Research (BCSIR), Dhaka, 1205 Bangladesh; 7 Biotechnology and Bioinformatics, Independent University, Dhaka, 1219 Bangladesh; 8 Biomedical Science Institute, Kyung Hee University, Seoul, 02447 Republic of Korea; 9 Department of Biomedical Science, Graduate School, Kyung Hee University, Seoul, 02447 Republic of Korea

**Keywords:** Cancer, Computational biology and bioinformatics, Biomarkers, Oncology

## Abstract

Recent therapeutic advances in breast cancer (BC) have improved survival outcomes; however, the prognosis for patients with bone metastasis (BM) remains poor. Hence, novel clinical biomarkers are needed to accurately predict BC BM as well as to promote personalized medicine. Here, we discovered a novel biomarker, TOR1B, for BM in BC patients via analysis of BC gene expression data and clinical information downloaded from open public databases. In cancer cells, we found high expression levels of *TOR1B* in the nucleus and endoplasmic reticulum. Regarding gene expression, the level of *TOR1B* was significantly upregulated in BC patients with BM (*p *< 0.05), and the result was externally validated. In addition, gene expression clearly demonstrated two distinct types of prognoses in ER- and PR-positive patients. In multivariate regression, the gene could be an independent predictor of BM in BC patients, i.e., a low expression level of *TOR1B* was associated with delayed metastasis to bone in BC patients (HR, 0.28; 95% CI 0.094–0.84). Conclusively, *TOR1B* might be a useful biomarker for predicting BM; specifically, patients with ER- and PR-positive subtypes would benefit from the clinical use of this promising prognostic biomarker.

## Introduction

Bone metastasis (BM) is the most devastating condition in patients with advanced breast cancer (BC), occurring in approximately 70% of patients, predominantly those with luminal A or estrogen/progesterone receptor‐positive (ER + /PR +) subtypes^1–3^. It seriously affects quality of life and survival outcomes through skeletal-related events (SREs), including bone pain, tumor-induced fracture, spinal cord compression, hypercalcemia and other complications^4,5^. The overall five-year survival rate for patients with BM is 22.8%, whereas for BC patients without BM, it is greater than 95%^6,7^. Although recent therapeutic advances in BC, such as targeted drugs and immunotherapy, have improved survival^8^, it is still unknown why some patients develop BM while others do not, even if they possess the disease phenomena. In most cases, metastatic lesions enter into a latent state that can last months, years, or even decades before forming a clinically detectable macrometastasis^1,9^. Numerous molecular markers may critically regulate the tumor microenvironment during the latency period, and the detection of tumors that are more prone to metastasis, particularly in bone, is vital^1,10,11^. Therefore, early detection of biomarkers may be crucial for the implementation of targeted therapeutic measures, eventually reducing the risk of skeletal complications and improving survival and quality of life.

A number of serum and tissue biomarkers have been extensively studied for the early diagnosis or prognosis of BM in BC patients. Integrin alpha 5 (ITGA5) is a mediator of breast-to-bone metastasis and a therapeutic target for treating osteolytic lesions in BC patients^12^. However, clinical trials have repeatedly failed to demonstrate the therapeutic benefits of integrin inhibitors in cancer patients^13^. Serum markers of bone formation (N-terminal propeptide of type-1 collagen [P1NP]) and bone resorption [C-telopeptide of type-1 collagen (CTX) and pyridinoline cross-linked carboxy-terminal telopeptide of type-1 collagen (1-CTP)] seem to have good prognostic value for bone-specific recurrence. Nevertheless, those markers have several limitations, such as poor sensitivity, small event numbers and lack of independent predictive ability^9^. Dedicator of cytokinesis protein 4 (DOCK4) is a promising prognostic biomarker for the early prediction of skeletal recurrence in BC. However, the DOCK4 expression level does not significantly influence overall survival in BC patients^14^. Other biomarkers, such as ICAM-1, cadherin-11, osteoactivin, bone sialoprotein, CCN3, IL-11, CCL2, CITED2, CXCR4, CTGF, OPN, CX3CR1, TWIST1, adrenomedullin and Enpp1, have also been identified as potential prognostic markers in BC with BM patients^7,15,16^. However, these biomarkers cannot be rationally used in clinical practice^17^. Therefore, additional novel useful biomarkers need to be identified and validated for the early prediction of BM in BC patients.

A previous study revealed that the Torsin/cofactor system has a functional relationship with nuclear envelope/nuclear pore complex (NPC) biogenesis^18^. Rodriguez-Bravo et al. molecularly established that NPC deregulation directly contributes to the progression of lethal prostate cancer and suggested a novel therapeutic target for prostate cancer^19^. A recent study characterized transcriptomic complexity with a personalized genomics approach in prostate cancer and identified TOR1A as the master regulator^20^. TOR1B and TOR1A are colocalized in the cell compartments retaining the same protein complex, demonstrating similar related functions^21^. A recent study discovered a higher level of TOR1B autoantigen in patients with pancreatic diseases, including pancreatic cancer^22^. However, the role of TOR1B in BC has not yet been determined. To investigate the relationship between TOR1B and BC BM, the present study aimed to identify the location of the protein in the cell and determine whether TOR1B has any prognostic value in BC BM, particularly in ER + BC, which is the most prone to developing BM.

## Results

### Subcellular localization and expression of TOR1B

**Figure 1:**
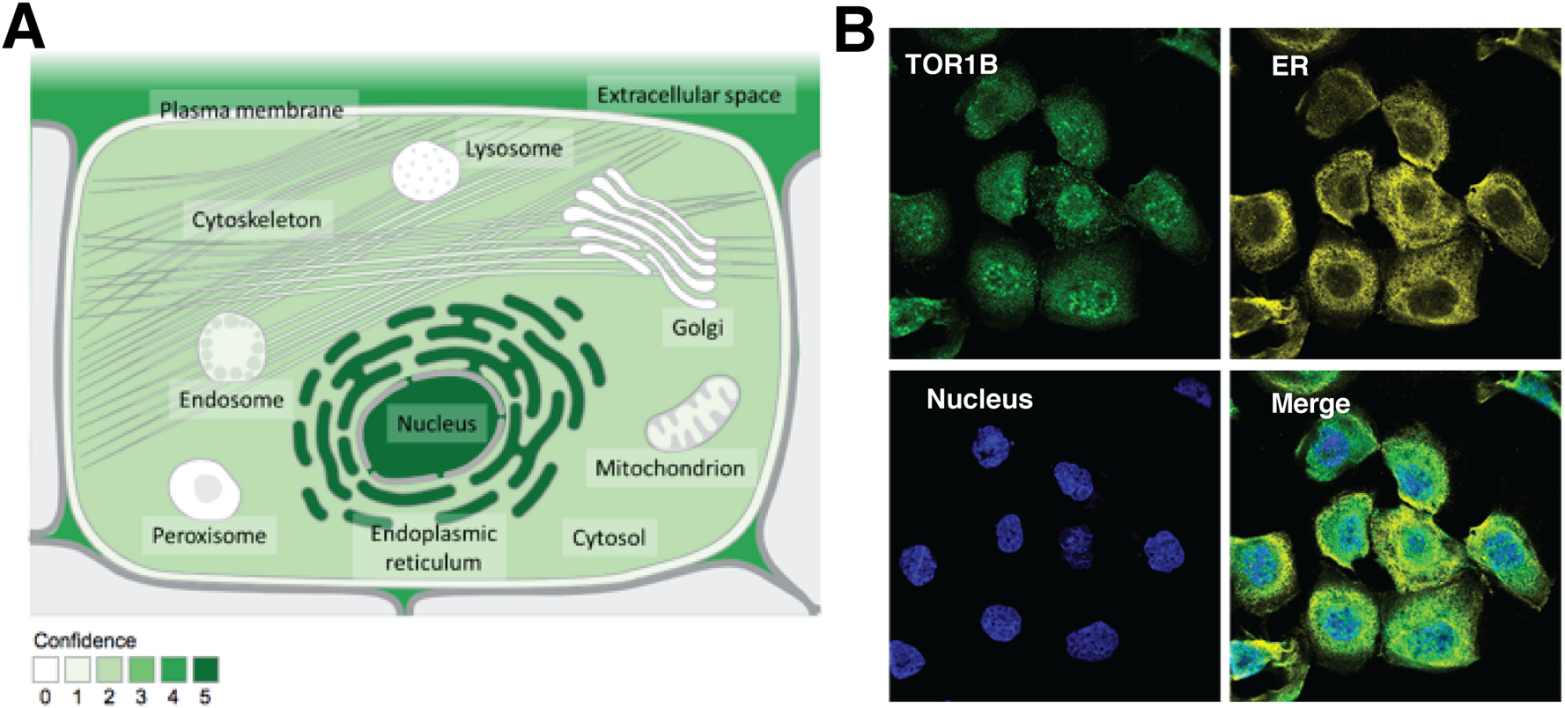
The localization of TOR1B at the cellular level. Subcellular locations from COMPARTMENTS (**A**) and the Protein Atlas (**B**). The confidence of each association is signified by stars, where ★★★★★ is the highest confidence and ★☆☆☆☆ is the lowest. All Image data shown were generated and downloaded with searching the queries from COMPARTMENTS (compartments.jensenlab.org) or Human Protein Atlas (www.proteinatlas.org, Protein Atlas version 22.0).

The subcellular localization of TOR1B was predicted using the COMPARTMENTS database by considering multiple information sources, i.e., database annotations, automatic text mining of the biomedical literature and sequence-based predictions regarding different cell types. The TOR1B protein product was identified with the highest confidence (confidence level 5) in the nucleus and endoplasmic reticulum and moderate confidence (confidence level 4) in the extracellular space (*p* value). The lowest confidence (confidence level 1) was in the cytosol, cytoskeleton, mitochondrion, peroxisome, endosome, lysosome and Golgi apparatus (Fig. 1A). TOR1B protein expression was projected using subcellular Atlas from the Human Protein Atlas database, and it was highly expressed in the nucleus and endoplasmic reticulum (Fig. 1B).

### TOR1B expression was upregulated in BC patients with BM

**Figure 2:**
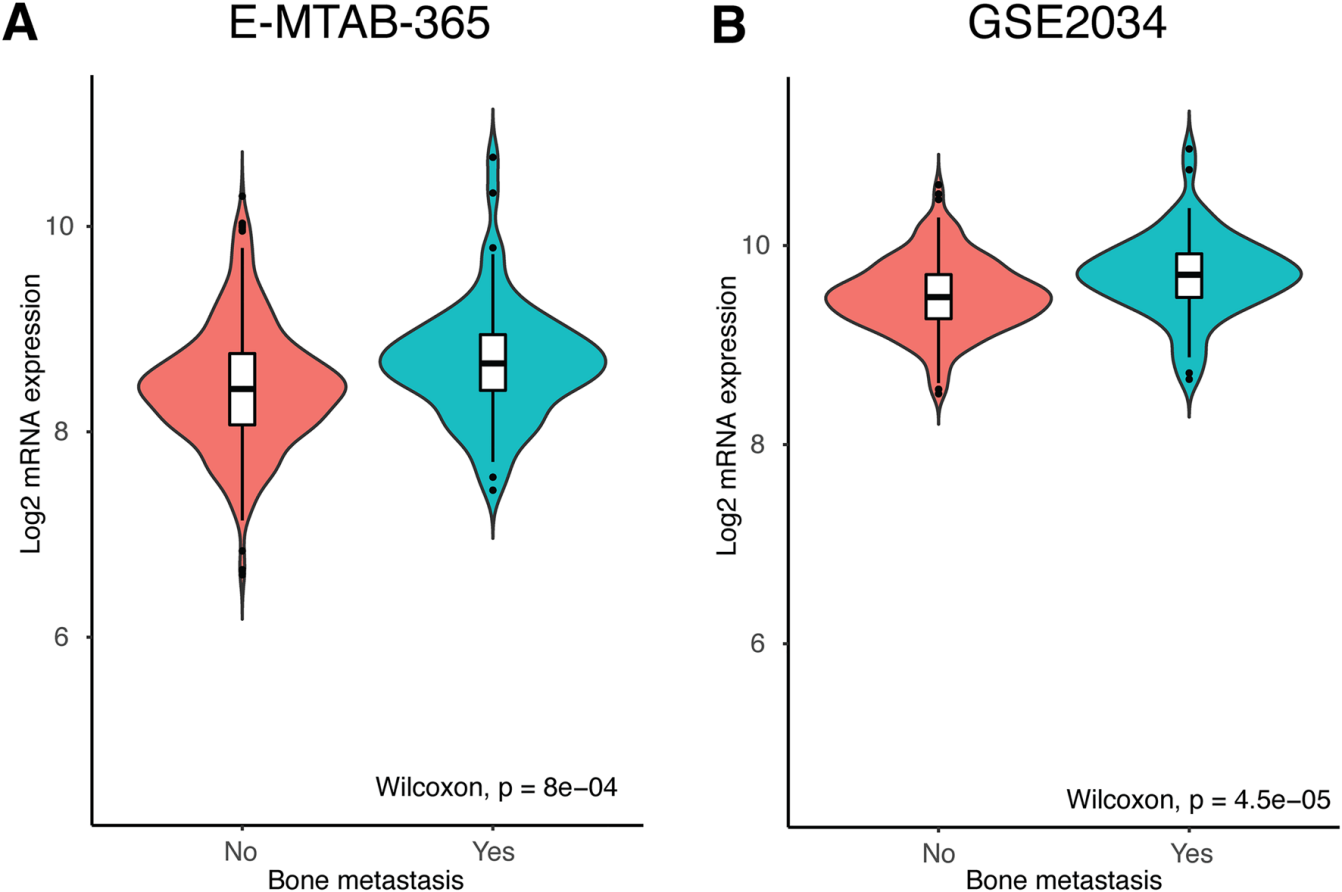
mRNA expression of *TOR1B* is significantly upregulated in bone metastatic patients in the E-MTAB-356 (**A**) and GSE2034 (**B**) datasets.

The mRNA expression of TOR1B was evaluated between metastatic and nonmetastatic BC patients in the E-MTAB-365 and GSE2034 datasets. The upregulation of TOR1B was highly significant in patients with BM (*p* = 8e-04 and *p* = 4.5e-05, metastatic vs. nonmetastatic BC, E-MTAB-365 and GSE2034 datasets, respectively) (Fig. 2). These results indicate that higher *TOR1B* expression at the transcriptional level may be associated with BM in BC patients.

### TOR1B expression and its correlation with clinicopathological features of BC

This study included 425 patients with BC from the E-MTAB-365 dataset and 286 patients from the GSE2034 dataset. The association of TOR1B expression with clinical features is shown in Table 1. Based on the median expression of *TOR1B,* patients in the E-MTAB-365 dataset were classified into groups: high expression (n = 212) and low expression (n = 213). The chi-square test was performed to study the association of clinicopathological features of BC patients with *TOR1B* expression (high and low). The results showed that the age and tumor grade of BC patients were not significantly correlated with TOR1B expression (*p* > 0.05). Regarding hormone receptor status, the presence or absence of HER2 was significantly associated with TOR1B expression (*p* = 0.008). Furthermore, TP53 mutation was highly correlated with TOR1B upregulation (*p* = 0.007). Remarkably, the CIT classification, TNM stage and BM of BC patients were significantly associated with TOR1B expression (*p *= 5.00E-04, *p *= 0.023 and *p *= 0.001, respectively). The clinicopathological characteristics of the BC patients are presented in Table 1.

**Table 1:** Clinicopathological features of breast cancer patients in two groups based on median expression of *TOR1B*.

Variable	Total	Low expression	High expression	*p* (χ2- test)
Number of patients (%)	425	213 (50.1)	212 (49.9)	
Age				
≤ 55	216 (51.3)	109 (25.9)	107 (25.4)	0.788
> 55	205 (48.7)	100 (23.8)	105 (24.9)	
ER				
No	103 (24.8)	50 (12)	53 (12.8)	0.74
Yes	312 (75.2)	158 (38.1)	154 (37.1)	
PR				
No	167 (40.3)	87 (21)	80 (19.3)	0.538
Yes	248 (59.8)	121 (29.2)	127 (30.6)	
HER2				
No	250 (85)	113 (38.4)	137 (46.6)	0.008
Yes	44 (15)	30 (10.2)	14 (4.8)	
Grade				
I	34 (8.2)	12 (2.9)	22 (5.3)	0.209
II	215 (51.5)	111 (26.6)	104 (24.9)	
III	168 (39.6)	86 (20.6)	82 (19)	
TP53 status				
Mutation	76 (50)	43 (28.3)	33 (21.7)	0.007
Wild type	76 (50)	60 (39.5)	16 (10.5)	
CIT				
basL	47 (11)	12 (2.8)	35 (8.2)	5.00E-04
lumA	81 (19)	49 (11.5)	32 (7.5)	
lumB	86 (20.2)	62 (14.6)	24 (5.6)	
lumC	62 (14.6)	34 (8)	28 (6.6)	
mApo	36 (8.5)	22 (5.2)	14 (3.3)	
normL	113 (26.6)	34 (8)	79 (18.6)	
TNM stage				
N0	134 (32.1)	79 (18.9)	55 (13.2)	0.023
N1	284 (67.9)	133 (31.8)	151 (36.1)	
Bone metastasis				
No	355 (83.5)	165 (38.8)	190 (44.7)	0.001
Yes	70 (16.5)	48 (11.3)	22 (5.2)	

### BM predictive ability of TOR1B in BC patients

**Figure 3:**
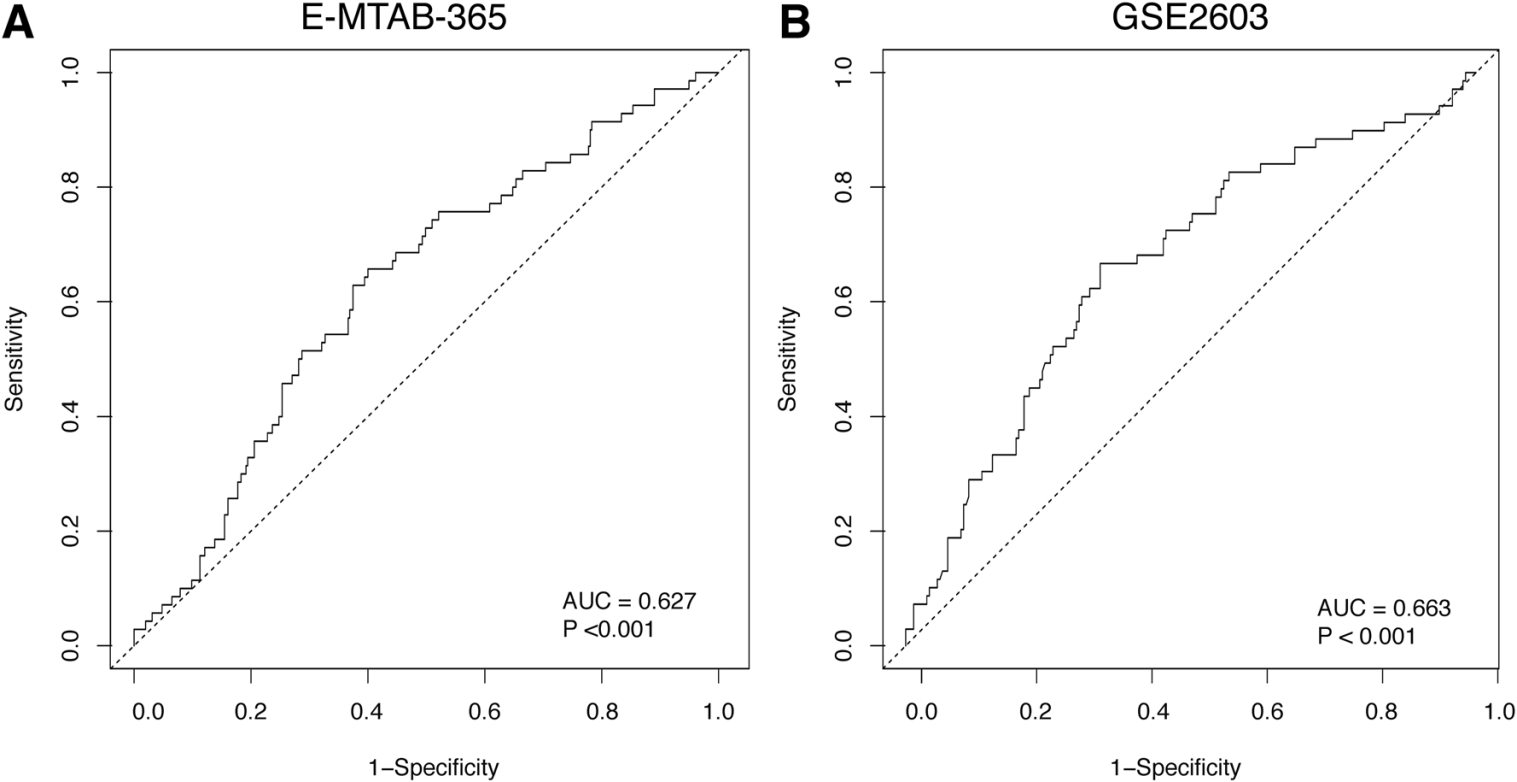
ROC curve analysis to test the validity of *TOR1B* gene expression in discriminating bone metastasis and non-bone metastasis status in breast cancer patients by ROC curve analysis for predicting bone metastasis in the E-MTAB-356 (**A**) and GSE2034 (**B**) datasets.

The sensitivity and specificity of TOR1B in predicting BM were evaluated from the area under the curve (AUC) of receiver operating characteristic (ROC) curve analysis. The AUC of TOR1B showed significant predictive capability for BM in BC patients in both the E-MTAB-365 and GSE2034 datasets (AUC = 0.627, *p *< 0.001 and AUC = 0.663, *p *< 0.001, respectively) (Fig. 3).

### High expression level of TOR1B was associated with poor outcomes in BM

**Figure 4:**
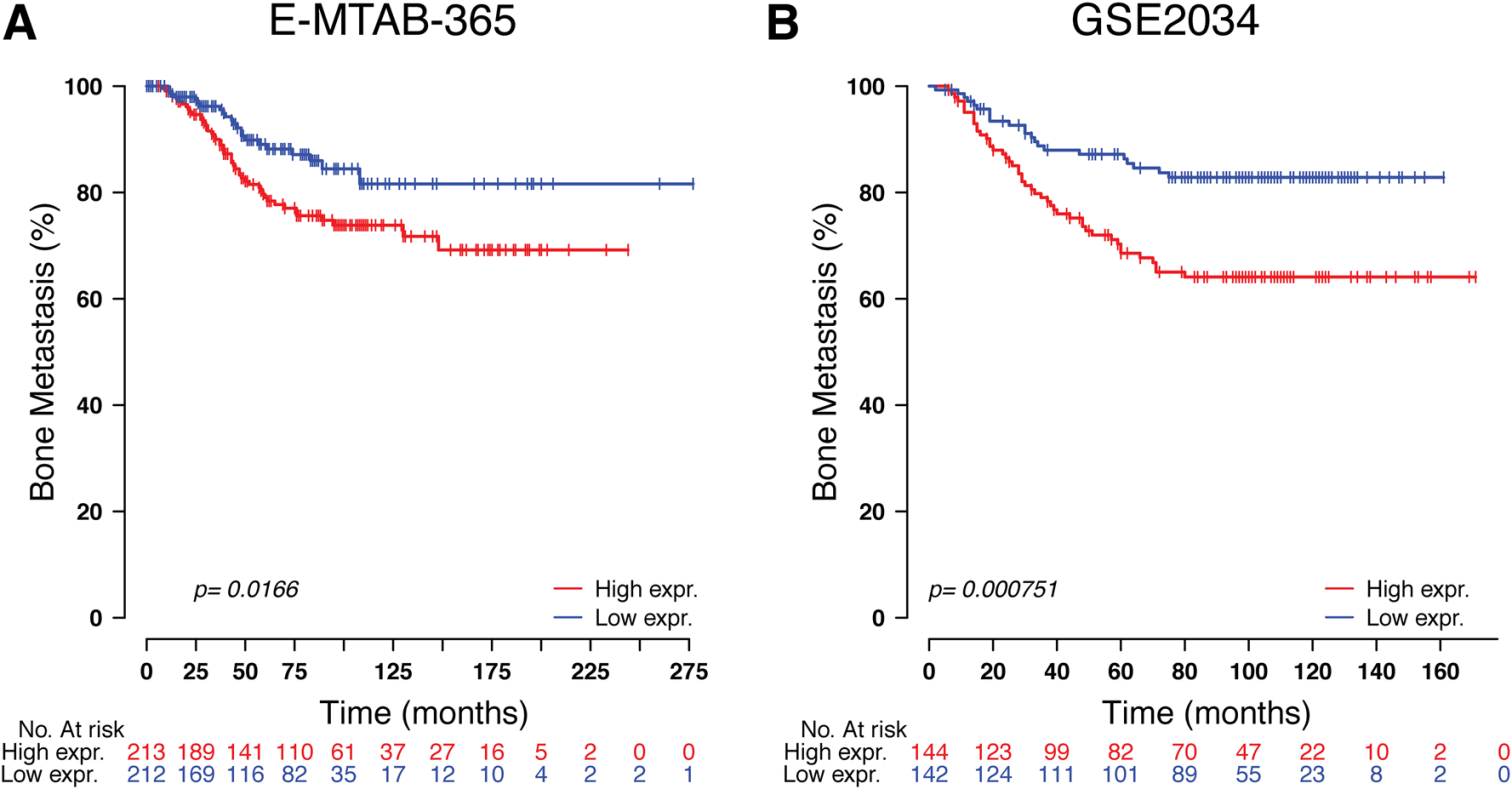
Kaplan‒Meier plots for bone metastasis of *TOR1B* expression. Kaplan‒Meier curve based on the expression of *TOR1B* in breast cancer patients from the E-MTAB-356 (**A**) and GSE2034 (**B**) datasets. The p value was calculated by log-rank tests.

**Figure 5:**
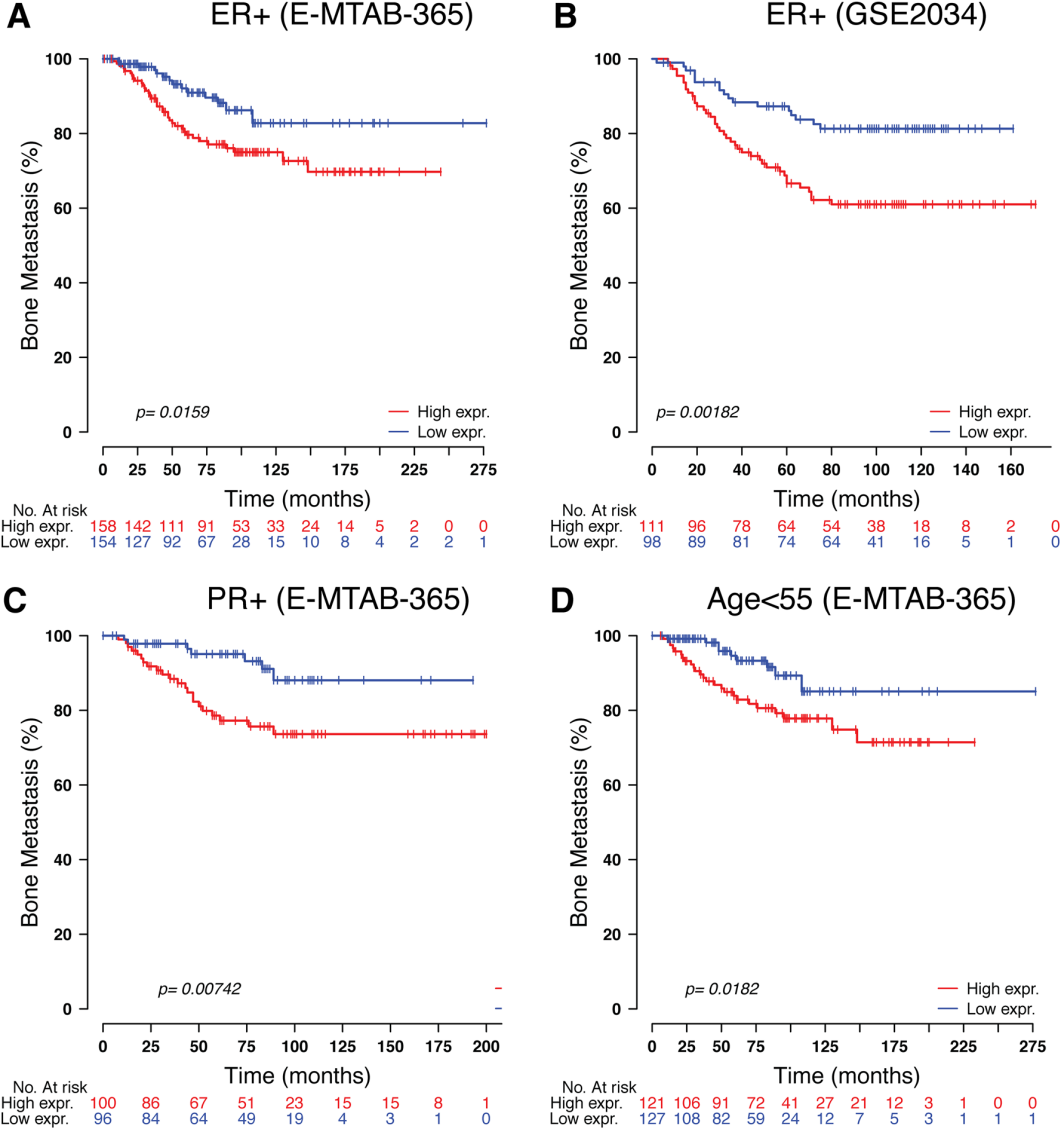
Significant association of *TOR1B* expression molecular markers and clinical information. Kaplan‒Meier curve based on the expression of *TOR1B* in ER-positive breast cancer patients from the E-MTAB-356 (**A**) and GSE2034 (**B**) datasets, PR-positive breast cancer patients (**C**) and patients aged more than 55 years (**D**) from the E-MTAB-356 dataset. Middle-aged groups of patients. The p value was calculated by log-rank tests.

**Figure 6:**
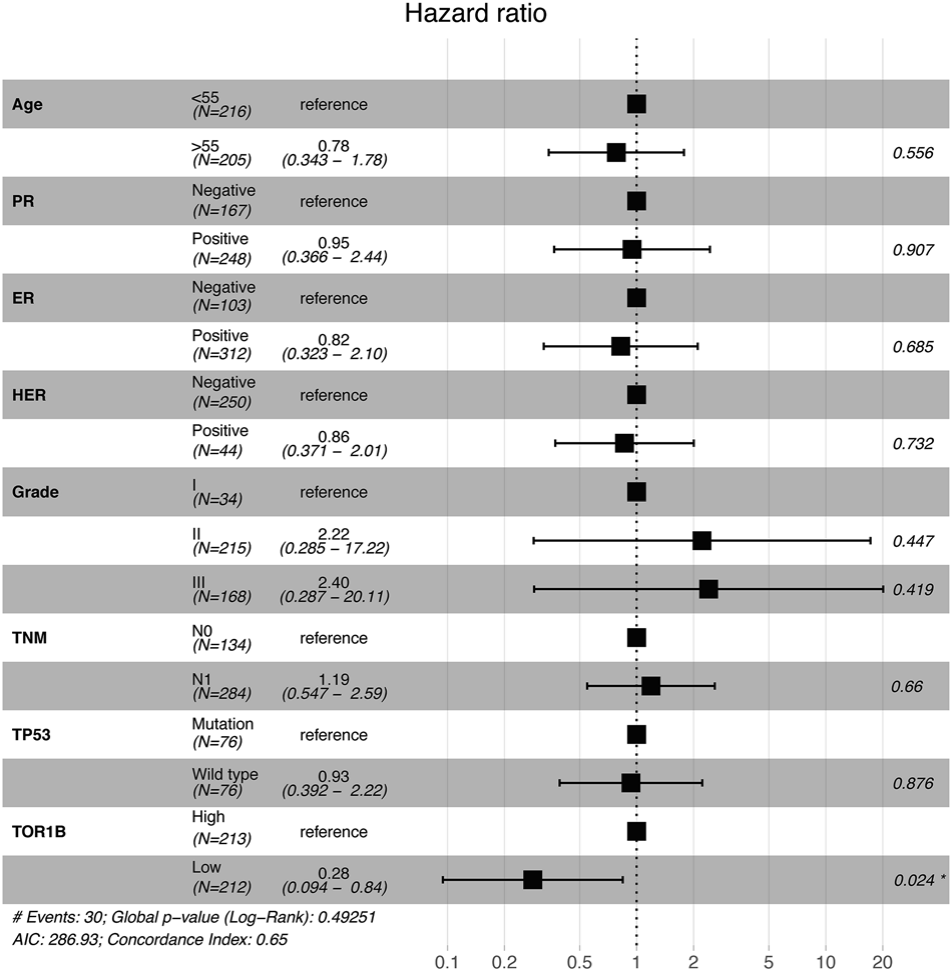
Multivariate Cox proportional hazard regression analyses of *TOR1B* and other clinical information.

To study the prognosis of BC patients with respect to TOR1B upregulation, we generated a Kaplan‒Meier plot, and the results showed that patients with upregulated TOR1B expression underwent earlier BM than patients with low TOR1B expression (Fig. 4). We further studied TOR1B expression in patients with BM using various clinicopathological features, i.e., age, presence or absence of hormone receptors, etc., to identify which patients were the most prone to develop earlier metastasis. The results demonstrated that *TOR1B* upregulation was significantly associated with earlier BM in ER + (E-MTAB-365, *p* = 0.0159 and GSE2034, *p* = 0.00182) and PR + (E-MTAB-365, *p* = 0.00742) BC, and patients who were younger than 55 years were more vulnerable (E-MTAB-365, *p* = 0.0182) to early metastasis (Fig. 5). Furthermore, to identify the prognostic predictor in BC patients, we performed a multivariate Cox proportional hazard regression analysis of *TOR1B* with other clinical information. The results revealed that *TOR1B* was an independent predictor of BM in BC patients (*p* = 0.024) (Fig. 6).

### TOR1B interaction network analysis

**Figure 7:**
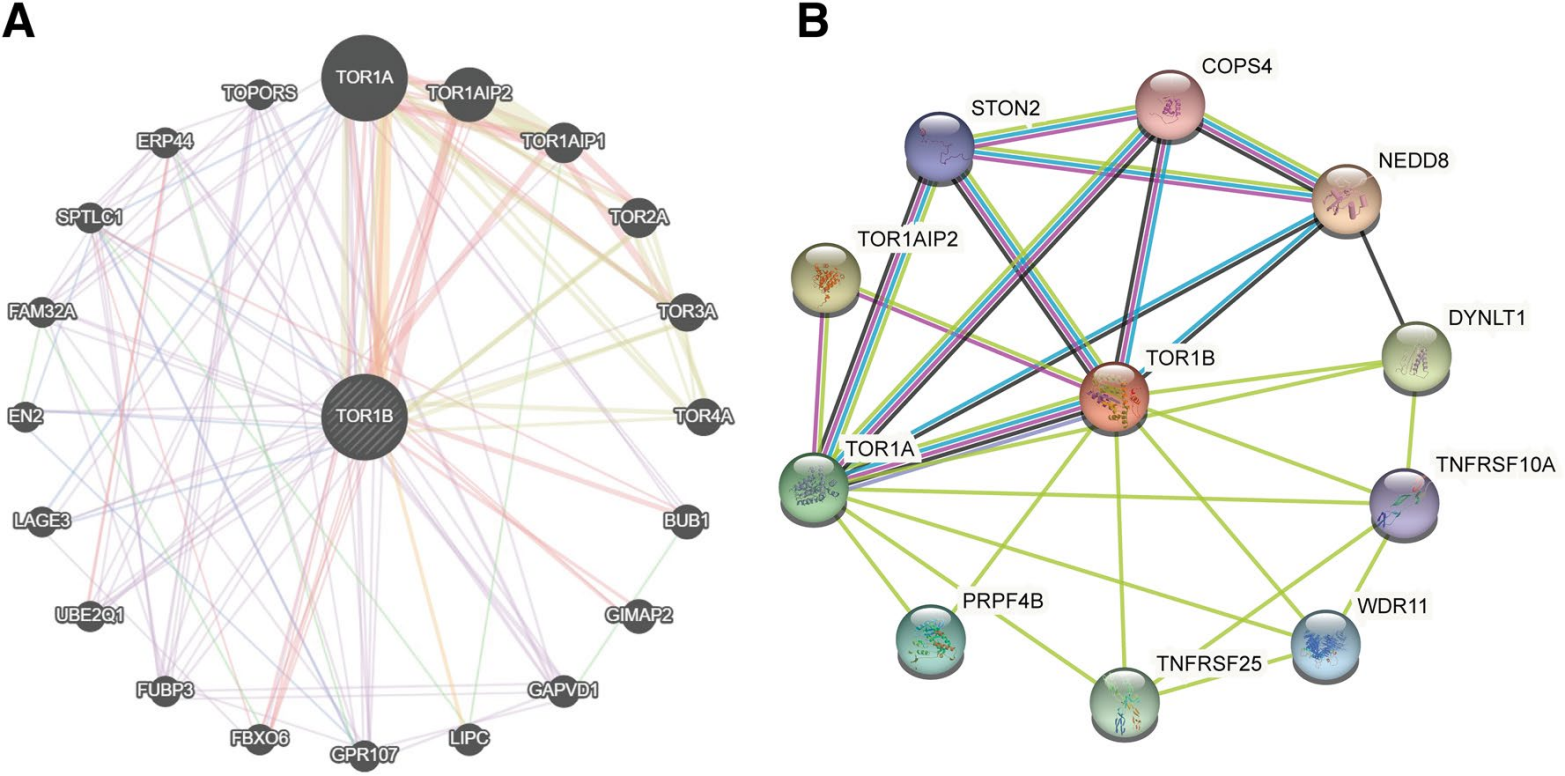
The interaction networks of *TOR1B* with other genes and proteins. (**A**) Interaction networks of *TOR1B* with other genes were generated using GeneMANIA. (**B**) Interaction networks of *TOR1B* with other genes were generated using STRING. All Image data shown were generated and downloaded with searching the queries from GeneMANIA (genemania.org) or STRING (string-db.org).

To study the interaction network of TOR1B with other proteins and genes, we performed a molecular network analysis by introducing TOR1B into the GeneMANIA and STRING databases. TOR1B was closely associated with other proteins, such as NEDD8, TOR1A1P2, DYNLT1, TOR1A, TNFRSF25, PRPF4B, WDR11, STON2, TNFRSF10A and COPS4 (Fig. 7). In addition, among the various *Homo sapiens* genes, TOR1B was related to the TOR1A, TOR1A1P2, TOR1A1P1, TOR2A, TOR3A, TOR4A, BUB1, GIMAP2, GAPVD1, LIPC, GPR107, FBXO6, FUBP3, UBE201, LAGE3, EN2, FAM32A, SPTLO1, ERP44 and TOPORS genes (Fig. 3).

## Discussion

Bone is the most frequent and common site for metastatic growth in BC patients. Once cancer cells have metastasized to the bone, they may have devastating impacts on cancer patients. There are several treatment approaches, such as hormone therapy, chemotherapy and bisphosphonate administration, while systemic therapies, including surgery, external radiation therapy and bone-targeted therapies, are used to treat BM^23^; however, a curative drug or treatment method has not yet been developed. Currently, multiple biomarkers have been identified for the prognostication of BM in BC patients^14,24,25^, which has improved therapeutic management; however, there is an unmet need to explore novel prognostic and predictive biomarkers. In this study, we identified and demonstrated TOR1B as a potential prognostic and predictive biomarker for BM in BC patients. TOR1B is an ATPase found primarily in the ER and nuclear envelope, and the protein product of the gene may act as a molecular chaperone assisting in the proper folding of secreted and/or membrane proteins and maintaining membrane integrity (https://www.proteinatlas.org/ENSG00000136816-TOR1B)^26^. Aggressive tumor microenvironments generated from diverse oncogenic, transcriptional and metabolic abnormalities disrupt ER homeostasis, leading to the aberrant activation of ER stress sensors, and the pathways of their downstream signaling may act as key drivers of tumor growth and metastasis, particularly failure to respond to therapies such as chemotherapy and targeted immunotherapy^26^. Therefore, enhancement of ER integrity and homeostasis by modulating TOR1B may provide novel therapeutic insight into metastatic BC patients. The protein is moderately available in the cytosol, which is also reflected in our study, and is highly similar to the neighboring gene TOR1A, which impairs the TGFβ signaling pathway in dystonia^27^. There are different variants or splices available for TOR1B that may have diverse functions, i.e., mutation of the protein (S38L variant) has a direct impact on lung squamous cell carcinoma (https://activedriverdb.org/mutation/show/NM_014506/38/L). Surprisingly, only a few functions of the protein have been discovered in which the crucial metabolic and immune functions are completely unknown yet (https://www.proteinatlas.org/ENSG00000136816-TOR1B/immune+cell).

Currently established BC prognostic markers, such as lymph node metastasis, primary tumor size, grade, etc., have several limitations^13,14,28^. Therefore, many women are overtreated and suffer severe side effects, as only 40% of patients develop recurrence and ultimately die due to metastasis, while 80% of them receive adjuvant chemotherapy^28^. The gene expression of primary and metastatic tumors has shown a similar pattern^29,30^, indicating that the differentially expressed genes between highly metastatic cells and their parental cells may only represent alterations caused by microenvironmental stimuli rather than their own gene expression pattern. Therefore, changes in differentially expressed genes in primary tumors are suggested as a powerful predictor that more accurately predicts the metastatic risk for individual BC patients^24,28,31–33^. In our previous study, we demonstrated 70 genes (92 probes) in late recurrence in primary BC patients, particularly those with lymph node-positive disease^33^. In the present study, we found overexpression of a single *TOR1B* gene in BC patients with BM. We robustly validated it in an external dataset. Based on high and low expression levels, the gene efficiently separated two subtypes of patients, those who developed BM and those who did not. Indeed, BC patients with high expression levels of TOR1B had early metastasis to bone compared to those with low expression levels of the gene. This finding is consistent with the previous study of Wang et al., who showed that patients with higher expression levels of the SRXN1 and TOR1B genes were not suitable for radiotherapy (https://doi.org/10.21203/rs.3.rs-1935488/v1). In fact, the gene can be used to predict a worse prognosis for BM in ER- and PR-positive BC patients, which is the distinct finding of our study, as TOR1B expression is only observed in overall BC without subtype specificities (https://www.proteinatlas.org/ ENSG00000136816-TOR1B/immune + cell). In addition, patients younger than 55 years of age were more prone to develop metastasis. This is also supported by the finding that increasing age at diagnosis is inversely associated with distant metastasis to bone and viscera^34^. The sensitivity and specificity of the prognostic marker showed satisfactory predictive capabilities (AUC 0.627 and 0.663) for BM in BC patients. This finding supports the meta-analysis of Zhao et al.by showing similar predictive ability in different studies of BC diagnostic accuracy^35^.

In both gene and protein interaction networks, the TOR1B gene has shown very close and strong associations with TOR1A, which impairs TGFβ signaling and generates multiorgan inflammation^36,37^. In humans, TGF-β acts as a metabolic driver in the tumor microenvironment to promote bone and lung metastasis via different mechanisms^38–40^. Therefore, exploration of the effect of TOR1B on TGF-β or other functional proteins might provide new therapeutic insight into BC metastasis.

In conclusion, we discovered *TOR1B* as a promising biomarker for predicting BM in BC patients, implying its prognostic value in the era of personalized medicine. We suggest that *TOR1B* might be used as a molecular diagnostic tool, particularly in ER + /PR + BC patients who are most prone to develop BM. Thus, we believe that *TOR1B* might be a helpful tool for orthopedic oncologists.

## Methods

### Subcellular localization of TOR1B

Subcellular localization of TOR1B was predicted and visualized by using the COMPARTMENTS database (http://compartments.jensenlab.org)^41^. All data were searched and filtered by selecting gene types from *Homo sapiens*, and the genes were further collected from different databases using unique gene symbols.

### Protein expression of TOR1B by immunohistochemistry

Protein expression levels of TOR1B were identified using the subcellular Atlas from the Human Protein Atlas database (https://www.proteinatlas.org/)^42^. Gene symbols were used to search for information from the database. Immunohistochemistry (IHC) staining of TOR1B in the endoplasmic reticulum and nucleus was assessed in the subcellular Atlas.

### Gene expression profiles, clinical data and analysis

Gene expression profiles and clinical information of BC patients were downloaded from the National Center for Biotechnology Information (NCBI) Gene Expression Omnibus (GEO) database (www.ncbi.nlm.nih.gov/geo/) and ArrayExpress (https://www.ebi.ac.uk/arrayexpress/). The robust multiarray average algorithm (RMA) was used to normalize the raw data to the median via Cluster 3.0. The GSE2034 and E-MTAB-365 datasets comprised 286 and 425 patients, respectively, and were taken as the training and validation cohorts. The probe set identifiers were transformed into gene symbols. The gene expression levels were normalized to the median center and were classified into two expression groups. Patients with TOR1B expression levels higher than the median were considered the high expression group, and patients with TOR1B expression levels less than or equal to the median were the low expression group. These groups were used for further analyses.

### TOR1B expression and its correlation with clinical information

A total of 711 patients were selected from the GSE2034 and E-MTAB-365 datasets to observe the mRNA expression levels of TOR1B among BM BC patients. Based on mRNA expression levels, the Kaplan‒Meier method was used to compare the survival between BC patients who developed BM and those who did not develop BM in total as well as those with ER- and PR-positive BC. Chi-square and log-rank tests were used to assess patient survival and metastasis.

### TOR1B gene‒gene and protein‒protein interaction network analysis

Interactions between the *TOR1B* gene and other genes were analyzed using the GeneMANIA database (https://genemania.org/)^43^. The gene interaction networks were built based on a functional association of genes present in the datasets, including protein and genetic interactions, signaling pathways, coexpression, colocalization and protein domain similarities. The analytical parameters were set according to the default indicators. Protein‒protein interaction (PPI) network analysis was performed using the Search Tool for the Retrieval of Interacting Genes/Proteins (STRING) database (http://www.string-db.org/)^44^. The PPI network of TOR1B was investigated in STRING’s *Homo sapiens* database. TOR1B protein networks were established based on the following eight criteria: experimental evidence and curated databases, gene neighborhood, gene fusion, gene co-occurrence, text mining, co-expression and protein homology.

### Statistical analysis

Kaplan‒Meier curves, chi-square tests and log-rank tests were performed using the R programming language (www.r-project.org). The Wilcoxon signed-rank test was used to compare the clusters between the two groups. The predictive capability of the biomarker was evaluated by multivariate analysis. The sensitivity and specificity of the prognostic biomarker were assessed by AUC. *p *< 0.05 was considered to be statistically significant.

## Data Availability

All the data analyzed in this study are publicly available and can be downloaded from the National Center for Biotechnology Information (NCBI) Gene Expression Omnibus (GEO) database (www.ncbi.nlm.nih.gov/geo/query/acc.cgi?acc=gse2034 for the dataset GSE2034) and ArrayExpress (www.ebi.ac.uk/biostudies/arrayexpress/studies/E-MTAB-365 for the dataset E-MTAB-365).
